# Maternal age and the risk of low birthweight and pre-term delivery: a pan-Nordic comparison

**DOI:** 10.1093/ije/dyac211

**Published:** 2022-11-09

**Authors:** Siddartha Aradhya, Anna Tegunimataka, Øystein Kravdal, Pekka Martikainen, Mikko Myrskylä, Kieron Barclay, Alice Goisis

**Affiliations:** Demography Unit and Department of Sociology, Stockholm University, Stockholm, Sweden; Centre for Economic Demography, Lund University, Lund, Sweden; Centre for Economic Demography, Lund University, Lund, Sweden; Department of Economics, University of Oslo, Oslo, Norway; Centre for Fertility and Health, Norwegian Institute of Public Health, Oslo, Norway; Max Planck Institute for Demographic Research, Rostock, Germany; Population Research Unit and Department of Social Research, University of Helsinki, Helsinki, Finland; Max Planck Institute for Demographic Research, Rostock, Germany; Population Research Unit and Department of Social Research, University of Helsinki, Helsinki, Finland; Demography Unit and Department of Sociology, Stockholm University, Stockholm, Sweden; Max Planck Institute for Demographic Research, Rostock, Germany; Swedish Collegium for Advanced Study, Uppsala, Sweden; Social Research Institute, University College London, London, UK

**Keywords:** Maternal age at birth, birth outcomes, sibling design, Nordic countries

## Abstract

**Background:**

Advanced maternal age at birth is considered a risk factor for adverse birth outcomes. A recent study applying a sibling design has shown, however, that the association might be confounded by unobserved maternal characteristics.

**Methods:**

Using total population register data on all live singleton births during the period 1999–2012 in Denmark (*N* = 580 133; 90% population coverage), Norway (*N* = 540 890) and Sweden (*N* = 941 403) and from 2001–2014 in Finland (*N* = 568 026), we test whether advanced maternal age at birth independently increases the risk of low birthweight (LBW) (<2500 g) and pre-term birth (<37 weeks gestation). We estimated within-family models to reduce confounding by unobserved maternal characteristics shared by siblings using three model specifications: Model 0 examines the bivariate association; Model 1 adjusts for parity and sex; Model 2 for parity, sex and birth year.

**Results:**

The main results (Model 1) show an increased risk in LBW and pre-term delivery with increasing maternal ages. For example, compared with maternal ages of 26–27 years, maternal ages of 38–39 years display a 2.2, 0.9, 2.1 and 2.4 percentage point increase in the risk of LBW in Denmark, Finland, Norway and Sweden, respectively. The same patterns hold for pre-term delivery.

**Conclusions:**

Advanced maternal age is independently associated with higher risk of poor perinatal health outcomes even after adjusting for all observed and unobserved factors shared between siblings.

Key Messages

- Advanced maternal age at birth is linked to an increased risk of adverse birth outcomes, but the evidence of whether the association is independent of maternal characteristics is inconclusive.
- Using total population registers from Denmark, Norway, Sweden and Finland, we test whether advanced maternal age at birth independently increases the risk of adverse birth outcomes using sibling models to reduce confounding by unobserved maternal characteristics shared by siblings.
- We employ a more careful application of the sibling design approach than prior studies.
- Advanced maternal age is associated with a higher risk of poor perinatal health outcomes even after adjusting for all observed and unobserved factors shared between siblings.

## Introduction

The postponement of childbearing has been one of the most prominent demographic developments in high-income countries in recent decades, with mean maternal age at childbearing rising to >30 years in the 2010s across most member countries of the Organization for Economic Co-operation and Development (OECD).^1^ This trend may have important spillover effects to other domains of life; advanced maternal age, typically defined as age ≥35 years, is considered a risk factor for poorer pregnancy and perinatal health outcomes,^2–6^ which have in turn been linked to lower cognitive ability and worse health in later life for offspring.^7–9^ At the same time, however, maternal age at childbearing reflects a variety of physiological and social processes, some of which may have positive implications for offspring welfare.^10–12^

Although most studies report that an advanced maternal age is associated with poor perinatal outcomes and pregnancy complications, whether this association is directly attributable to the consequences of reproductive ageing, or some combination of factors that confound the association between maternal age and perinatal outcomes, is not yet clear. A recent study has cast doubt upon whether maternal age is an independent or important determinant of perinatal health outcomes.^13^ Comparing siblings born to the same mother at different ages using Finnish population register data, Goisis *et al.*^13^ reported that maternal age is not independently associated with the risk of low birthweight (LBW) and pre-term birth (PTB). This finding suggests that unobserved factors that are shared by siblings in the same family—such as pre-existing maternal medical conditions and socio-demographic characteristics—may confound the relationship between maternal age at birth and perinatal outcomes. This finding is consistent with other studies that have found that negative effects of advanced maternal age are substantially attenuated after adjusting for socio-demographic and socio-economic disadvantage.^14^

The findings reported by Goisis *et al.*^13^ demand further investigation and a careful assessment of potential generalizability. In this study, we investigate the relationship between maternal age at childbirth and the risk of LBW and PTB using population register data from Denmark, Finland, Norway and Sweden, and an improved empirical approach that avoids the collinearity between maternal age and birth year^15^ that limits the interpretation of the results reported in Goisis *et al.*^13^ These four Nordic countries are ideal for a comparative analysis for several reasons: (i) each collects administrative register data with almost identical quality and coverage; (ii) all four share important similarities in institutional and public health systems; and (iii) the Nordic countries are at the vanguard of the secular fertility postponement trend, with the mean age at birth >30 years.^1^

## Data

In this study, we use population register data from Denmark, Finland, Norway and Sweden covering birth cohorts from 1999 to 2012 in Denmark, Norway and Sweden, and birth cohorts from 2001 to 2014 in Finland. All data are drawn from each country’s Medical Birth Registers, including information on the linked children and mothers. Each of these data sets is considered to be of exceptionally high quality.^16–18^ In each country, we identify live, singleton births.

We access the Danish Medical Birth Register via the Danish Health Data Authority through Statistics Denmark (*N* = 580 133). We access the Finnish Medical Birth Register through the National Institute for Health and Welfare of Finland (*N* = 568 026). Access to the Norwegian Medical Birth Register is provided by the Norwegian Institute of Public Health (*N* = 540 890). We access the data for Sweden through the Swedish Interdisciplinary Panel, administered by the Centre for Economic Demography at Lund University (*N* = 941 403). Our data sources for Finland, Norway and Sweden provide data on all births recorded in the birth cohorts that we study. The Danish data that we have access to are a 90% random sample of the full population.

Observations with missing values for LBW or PTB were dropped (Denmark: 8122 or 1.4%; Finland: 2352 or 0.3%; Norway: 6138 or 0.7%; Sweden: 7227 or 0.6%). Multiple births were excluded from the analyses (Denmark: 32 505 or 3.4 %; Finland: 23 585 or 2.9%; Norway: 28 380 or 3.5%; Sweden: 32 370 or 2.7%), because multiple births have higher risks of LBW and PTB, and are more common at later maternal ages.^19^ Since sibling fixed-effects models are estimated using the variation between siblings, it was necessary to exclude children without siblings.

### Birth outcomes

We study two binary birth outcomes: whether a child was born with LBW (<2500 grams at birth) and whether the child was delivered with PTB (<37 weeks of gestation).

### Maternal age at birth

The main explanatory variable was maternal age at childbirth. We divided maternal age into the following categories: <18, 18–19, 20–21, 22–23, 24–25, 26–27, 28–29, 30–31, 32–33, 34–35, 36–37, 38–39, 40–41 and 42+ years. This categorical approach allows us to capture potential non-linearities in the association between maternal age and LBW and PTB. The age group 26–27 years is used as the reference category.

### Control variables

The sibling fixed-effects model adjusts for all time-invariant observed or unobserved factors that are shared by siblings. In terms of time-varying characteristics that vary from sibling to sibling, we included controls for parity (1, 2, 3, 4+) and child’s sex. We control for parity because within families later births always occur at higher maternal ages and births at parities two and above have a lower risk of LBW than first births.^20^^,^^21^ Moreover, by adjusting for parity, we account for other unobserved factors that vary between siblings by birth order, such as smoking during pregnancy.^22^^,^^23^

## Methods

### Statistical analysis

We estimate the association between maternal age and birth outcomes by fitting three different linear probability models (LPMs) for each of our two binary outcomes for each country separately, with fixed effects specified at the level of the sibling group. We compare siblings born to the same mother at different ages. This approach allows us to adjust for all unobservable maternal factors that are shared between siblings, including social background, pre-childbearing health behaviours, maternal age at first birth, unobserved aspects of maternal health and some genetic factors. Sibling fixed-effects models do not, however, adjust for factors that vary between siblings unless they are explicitly modelled.

We rely on LPMs over nonlinear models such as the logit specification because only the former allow direct comparisons of coefficients across models and groups,^24^ and that is a specific aim of our study. Average marginal effects from logit models are comparable, but are then close to unstandardized coefficients from LPMs.^25^ The LPM is a consistent estimator even for binary outcomes;^25^ our data are very large and with heteroskedasticity robust standard errors, the often-cited inference problem due to heteroskedastic residuals in the LPM is mitigated. Furthermore, LPMs have been shown to outperform conditional logistic models when the outcome is rare (specifically <25% of occurrences).^26^

Model 0 is a bivariate fixed-effects model, estimating the association between LBW, PTB and maternal age in the absence of any further control variables. Model 1 controls for parity (1, 2, 3, 4+) and child sex. Model 2 introduces an additional control for year of birth using individual-year dummy variables and is comparable to the main estimation model presented in Goisis *et al.*^13^ As previous work has highlighted, maternal age and birth year are collinear in a sibling comparison model^15^ and Model 2 is therefore primarily included for illustrative purposes. Model 1 is our preferred model for identifying the effects of maternal age on LBW and PTB.

### Sensitivity analyses

We conducted two sensitivity analyses. First, in Model 3, we estimate sibling fixed-effects models adjusting only for sex in order to investigate the influence of parity on the model estimates (specifically the change from Model 0 to Model 1). Second, we estimate Model 4, adjusting for inter-pregnancy interval (IPI) in addition to sex and parity. IPI is included as a categorical variable for the number of months between pregnancies for Finland and Norway (month of birth was not provided with the Danish or Swedish data deliveries). This exercise was done since IPI and maternal age are associated and IPI or its determinants (such as maternal health/behaviours or living conditions) may affect the outcome.^27^ Although research has shown that in within-family analyses only very short IPIs (<9 months) impact perinatal outcomes in high-incomecontexts,^28–30^ it is worth demonstrating the maternal age effect independent of IPI. Moreover, IPIs of <9 months are infrequent in the contexts studied. We estimate these models by implementing the method proposed by Kravdal to include the IPI in families with two or more siblings.^31^^,^^32^

## Results

Table 1 presents the descriptive characteristics of the populations. Supplementary Figures S1–S3 (available as Supplementary data at *IJE* online) show trends in maternal age at childbearing and fertility across the four countries.

**Table 1:** Characteristics of the study population by country, children born during the period 1999–2012 in Denmark, Norway and Sweden, and during the period 2001–2014 in Finland

			Country			
Variable	Category	Summary statistic	Denmark	Finland	Norway	Sweden
Low birthweight		%	3.30	3.03	3.45	3.18
Pre-term birth		%	4.80	4.36	5.28	5.40
Maternal age (years)	<18	%	0.34	0.38	0.19	0.17
	18–19	%	1.44	1.93	1.16	0.86
	20–21	%	3.17	4.69	3.31	2.78
	22–23	%	5.72	7.22	5.78	5.35
	24–25	%	9.89	10.35	9.03	8.60
	26–27	%	14.64	13.45	12.63	12.35
	28–29	%	17.33	15.12	15.42	15.13
	30–31	%	16.49	14.44	15.26	16.06
	32–33	%	13.04	12.00	13.73	14.22
	34–35	%	8.87	8.84	10.38	10.70
	36–37	%	5.15	5.80	6.65	6.94
	38–39	%	2.57	3.33	3.63	3.83
	40–41	%	0.99	1.61	1.61	1.85
	42+	%	0.35	0.84	0.72	1.17
Parity	1	%	37.68	34.41	36.65	32.75
	2	%	42.97	38.57	41.91	42.54
	3	%	14.43	15.61	15.79	16.85
	4+	%	4.92	11.42	5.66	7.86
Sex	Male	%	51.37	51.24	51.43	51.45
	Female	%	48.63	48.76	48.57	48.55

*N*			580 133	568 026	540 890	941 403

**Figure 1:**
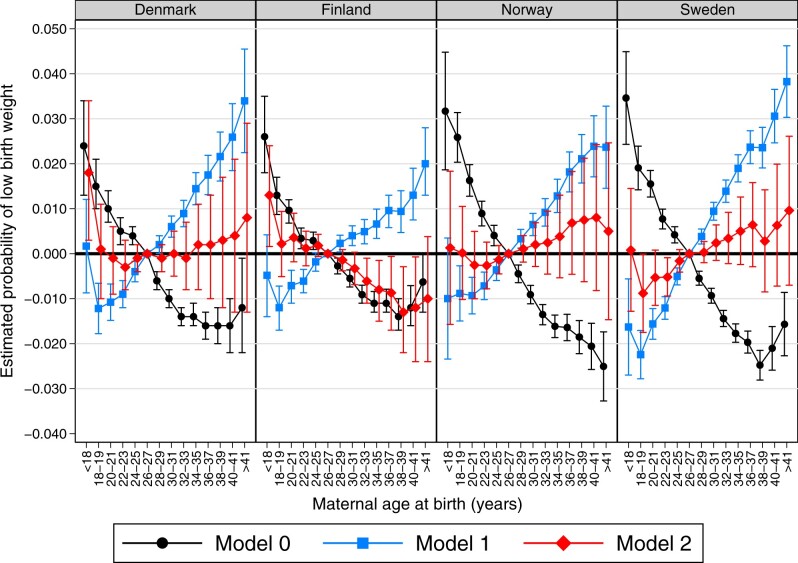
Results from linear probability models using sibling fixed effects corresponding to Models 0, 1 and 2 for the probability of low birthweight by maternal age category (relative to age 26–27 years) for children born from 1999 to 2012 in Denmark, Norway and Sweden, and from 2001 to 2014 in Finland. Error bars are 95% CIs. Model 0 is a bivariate model; Model 1 controls for child sex and parity; Model 2 controls for child sex, parity and birth year. Full table of results is shown in Supplementary Table S1 (available as Supplementary data at *IJE* online)

Figure 1 displays the results for the association between maternal age and the risk of LBW for Denmark, Sweden, Norway and Finland, respectively, for Models 0, 1 and 2, estimated from sibling fixed-effects models. The results from Model 0 show that the probability of LBW decreases with maternal age in all four countries. Model 1 introduces a control for parity. The results from Model 1 show that the risk of LBW increases with maternal age across all four countries. For example, mothers aged 22–23 years display 0.9 (β_Denmark_: –0.009; 95% CI: –0.012, –0.006), 0.6 (β_Finland_: –0.006; 95% CI: –0.009, –0.004), 0.7 (β_Norway_: –0.007; 95% CI: –0.010, –0.004) and 1.2 (β_Sweden_: –0.012; 95% CI: –0.015, –0.010) percentage-point lower risks of LBW relative to the reference category in the respective countries. This corresponds to ∼27%, ∼20%, ∼20% and ∼38% lower risks of LBW relative to the baseline probability in each country, respectively. On the other hand, mothers aged 38–39 years displayed 2.2 (β_Denmark_: 0.022; 95% CI: 0.016, 0.027), 0.9 (β_Finland_: 0.009; 95% CI: 0.005, 0.014), 2.1 (β_Norway_: 0.021; 95% CI: 0.016, 0.026) and 2.4 (β_Sweden_: 0.024; 95% CI: 0.019, 0.028) percentage-point higher risks of LBW relative to the reference category in Denmark, Finland, Norway and Sweden, respectively, corresponding to 66%, 30%, 61% and 75% higher risks of LBW relative to the baseline probability in each country.

The results from Model 2 are shown for illustrative purposes. The results from Model 2 show an attenuated gradient in all four countries and a decrease in precision leading to a lack of differences between most of the age groups relative to the reference group. However, due to perfect collinearity between maternal age and birth year in a sibling comparison model, it is not possible to separate the relative contribution of each factor to the risk of LBW.

**Figure 2:**
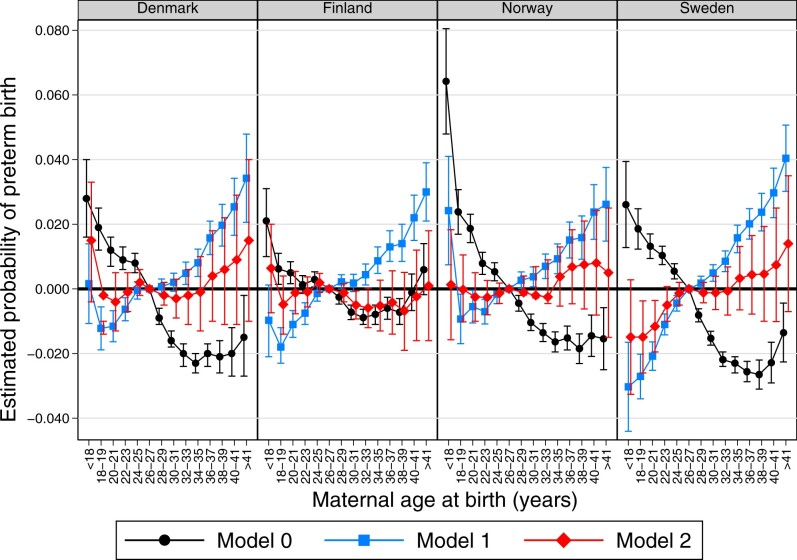
Results from linear probability models using sibling fixed effects corresponding to Models 0, 1 and 2 for the probability of pre-term birth by maternal age category (relative to age 26–27 years) for children born from 1999 to 2012 in Denmark, Norway and Sweden, and from 2001 to 2014 in Finland. Error bars are 95% CIs. Model 0 is a bivariate model; Model 1 controls for child sex and parity; Model 2 controls for child sex, parity and birth year. Full table of results is shown in Supplementary Table S2 (available as Supplementary data at *IJE* online)

The results from analyses examining PTB are presented in Figure 2. The results from Model 0 generally show a monotonic decline in the probability of PTB as maternal age increases from <18 to 30 years. After age 30 years, the probability of PTB plateaus in Denmark and Norway, but in Finland and Sweden it continues to decrease before rising again at age ≥40 years.

The results from Model 1 for PTB are similar to the results for LBW, with a monotonic increase in the probability of PTB with increasing maternal age across all four countries, with the exception of births to mothers <18 years of age in Norway, Denmark and Finland. Mothers aged 22–23 years of age display 0.6 (β_Denmark_: –0.006; 95% CI: –0.010, –0.003), 0.8 (β_Finland_: –0.008; 95% CI: –0.011, –0.004), 0.7 (β_Norway_: –0.007; 95% CI: –0.011, –0.003) and 1.1 (β_Sweden_: –0.011; 95% CI: –0.014, –0.008) percentage-point lower risks of experiencing a PTB compared with the reference group in Denmark, Finland, Norway and Sweden, respectively. This corresponds to 13%, 17%, 13% and 20% lower risks of PTB relative to the baseline probability in each country, respectively. An elevated risk of PTB was observed among mothers older than the reference category. For example, mothers aged ≥38–39 years experience 2.0 (β_Denmark_: 0.020; 95% CI: 0.013, 0.026), 1.4 (β_Finland_: 0.014; 95% CI: 0.009, 0.020), 1.6 (β_Norway_: 0.016; 95% CI: 0.009, 0.023) and 2.4 (β_Sweden_: 0.024; 95% CI: 0.018, 0.095) percentage-point higher risks of PTB in Denmark, Finland, Norway and Sweden, respectively. This corresponds to 41%, 32%, 30% and 44% increases relative to the baseline probability, respectively. The Model 2 results are once again shown for illustrative purposes and, in line with the results for LBW, they show that there is an attenuated gradient and a loss of precision of the estimates.

The sensitivity analyses examining the association between maternal age and LBW and PTB adjusting only for sex highlight the important role that the birth order adjustment plays (Supplementary Table S2, Model 3, available as Supplementary data at *IJE* online). Specifically, model estimates from these sensitivity analyses were nearly identical to those from Model 0. Similarly, results from sensitivity analyses adjusting for IPI in Norway and Finland only slightly weakened the relationship between maternal age and the risk of LBW, and had no impact on the relationship between maternal age and PTB (Supplementary Table S2, Model 4, available as Supplementary data at *IJE* online).

## Discussion

In this study, we used a sibling comparison design to examine whether maternal age was associated with the probability of LBW and PTB in Denmark, Finland, Norway and Sweden. The results from our preferred model (Model 1) show a clear and substantial positive association between maternal age and the probability of both LBW and PTB. These results were obtained after controlling for parity, but not for birth year.

Our study builds upon Goisis *et al.*^13^ and their novel application of sibling fixed-effects models to study the association between maternal age and perinatal outcomes, but our estimates are inconsistent with their reported findings. Goisis *et al.*^13^ reported that there was no meaningful association between maternal age and LBW and PTB after holding constant factors shared by siblings. A potential explanation for the inconsistent findings reported in Goisis *et al.*^13^ may be an overlooked problem in the empirical approach employed. The within-family model preferred by Goisis *et al.*^13^ included variables for both maternal age at birth and the child’s year of birth. In a sibling comparison model, maternal age at birth and year of birth are perfectly collinear, meaning that the effect of maternal age cannot be identified.^15^^,^^33^ This is illustrated in Model 2 in this study.

Our preferred estimation model (Model 1) includes controls for parity and child sex, but not birth year due to the collinearity between maternal age and birth year when comparing births to the same mother. By excluding a control for birth year, our models are estimating the joint effect of maternal age and birth year. We would prefer to obtain estimates for maternal age net of the effect of birth year, but this is not possible when applying the sibling comparison design. Although we have restricted our analyses to a period over which the prevalence of LBW and PTB hardly varied in the Nordic region (see Supplementary Figure S4, available as Supplementary data at *IJE* online), we nevertheless cannot be certain about the extent to which our results are being driven by birth year. In addition, the results from Model 1 highlight the important role of parity adjustments when estimating the effect of maternal age on LBW and PTB. Since first-born children experience an elevated risk of LBW and PTB and occur, on average, at younger maternal ages, the maternal age association with LBW and PTB becomes positive in sibling fixed-effects models after adjustment for parity; in fact, the maternal age association entirely reverses in comparison to Model 0, which indicates that the net beneficial effect of higher parity or the time-varying processes it reflects more than outweighs the net detrimental effect of increasing maternal age on birth outcomes. Nevertheless, it is the net effect of maternal age that is of interest to couples planning when to have a child of a certain birth order or to those advising on such matters. Note also that the beneficial effect of higher birth order is consistent with the existing research showing large parity differences in perinatal health.^20–22^

Although some previous work has suggested that the association between advanced maternal age and poor maternal outcomes is driven by socio-economic disadvantage or other unobserved factors shared by siblings,^13^^,^^14^ the results from our study are more consistent with the hypothesis that advanced maternal age increases the probability of LBW and PTB through physiological pathways. Reproductive ageing, manifested through the accumulation of DNA damage in germ cells,^34^ declines in oocyte quality,^35^ weakening of the placenta,^36^ and pregnancy complications such as gestational hypertension^37^ likely drive the association between maternal age and both LBW and PTB. It is worth noting that since maternal and paternal age are highly correlated, our results may also reflect the negative impacts of paternal reproductive ageing. Reproductive ageing of fathers is also associated with perinatal outcomes, e.g. through sperm abnormalities or chromosomal mutations that may affect fetal growth.^38–40^

The results from our study complement previous work by Lawlor and colleagues^41^ who use a cousin fixed-effects design (which may also be described as grandmother fixed effects) to study the association between maternal age and perinatal outcomes for first-born children. The sibling and cousin fixed-effects designs have different strengths and weaknesses with respect to the generalizability of findings. On the one hand, the cousin fixed-effects design allows the inclusion of ‘only children’, whereas the sibling fixed-effects approach conditions on mothers who have at least two children; including only children increases the external validity of the findings because ∼15% of women in the Nordic countries that we study have one child.^1^ Note, however, that the cousin comparison approach introduces other considerations with respect to external validity. First, offspring whose mothers do not have any siblings would be excluded from a cousin fixed-effect analysis, as would offspring whose mothers do not have any sisters. Second, although the cousin fixed-effect design may include only children, one may wish to compare the birth outcomes of only one child per mother (e.g. first births) so as to avoid combining the cousin and sibling comparisons, which would also reduce generalizability. The sibling fixed-effects design arguably has greater internal validity because it adjusts for all time-invariant observed and unobserved characteristics of the mother (e.g. family socio-economic conditions during the mother’s childhood as well as educational and occupational status in adulthood and any underlying maternal health conditions), which may be particularly important for understanding the association between maternal age at birth and both LBW and PTB. Despite these differences, the two approaches provide highly similar results. We replicate the analyses using a cousin FE design as in Lawlor *et al.*^41^ and the results (presented in Supplementary Figure S5, available as Supplementary data at *IJE* online) are highly similar to those presented in the main text.

This study has several strengths. First, we use total population data of equally high quality and coverage from four Nordic countries, each of which allows us to identify siblings. Second, the data are not prone to self-selection since they come from administrative population registers. Third, we are able to account for all unobserved maternal characteristics that are shared between siblings. To the best of our knowledge, no previous study has been able to analyse such a large geographic region with this degree of precision.

This study also has limitations specifically attributable to the sibling fixed-effects model that we employ. First, our analyses are based upon a subset of women who had at least two live births, because the sibling fixed-effects model exploits variation between siblings. Although the fixed-effects models reduce confounding from unobserved factors shared by siblings, the exclusion of one-child sibling groups means that we cannot generalize our results to this population. However, our results show that advanced maternal age increases the probability of LBW and PTB, and we have no *a priori* reason for believing this should be different for women who have only one child. Furthermore, research using cousin comparisons that adjust for some family characteristics but include only children produce similar results.^41^ Most women had two or more children in the country cohorts that we study^1^ and therefore our results are generalizable to the majority of the population in the Nordic region. While the results from analyses that employ sibling comparisons differ substantially from analyses that do not due to unobserved confounding, we prefer the sibling comparison models for the benefits of improved statistical identification.

Second, the sibling comparison does not adjust for all factors that may vary over time within the family, such as changes between births in maternal health status, socio-economic status and health behaviours.^42^ Although controlling for parity does partially adjust for changing family circumstances between births, we cannot exclude the possibility of unobserved time-varying confounding.^43^

Third, it is known that sibling model estimates can be severely biased if the outcome for one child affects subsequent fertility and thus the maternal age ‘exposure’ for a younger sibling.^44^ However, this is most likely to be important if the outcome is child death.^31^ LBW and PTB are likely to have a smaller impact on subsequent fertility. A separate potential carryover effect can occur if e.g. the perinatal health of the first child triggers behavioural changes in the mother that reduce the likelihood of adverse birth outcomes occurring in subsequent births (e.g. smoking during pregnancy).^42^ Nonetheless, this type of confounding would lead to a downward bias of our estimates.

The mean age at childbearing has risen steadily across high-income countries since the 1970s, with the mean age at first birth nearing age 30 years across high-income countries and even exceeding age 30 years in countries such as Italy, Japan, Spain and Switzerland. This study sheds additional light on the potential public health consequences of this secular demographic trend. In contrast to recent work, our study suggests that childbearing at older maternal ages does increase the risk of LBW and PTB. Moreover, the consistency of our results across these four countries suggests that it may be reasonable to generalize this finding to other countries with similar fertility regimes and welfare systems.

## Ethics approval

Ethical approval for the Danish data was provided by Statistics Denmark (Project FAMC706980). Data access to the Danish data was provided through a collaboration between the Centre for Economic Demography, Lund University and The Danish Institute for Human rights. Ethical approval for the Finnish data was provided by Statistics Finland (TK-53–1121-18) and Findata (THL/2179/14.02.00/2020). Ethical approval for the use of the Norwegian data was obtained from the Regional Committees for Medical and Health Research Ethics (approval #2013/2394). Ethical approval for the Swedish data analysis was approved by the Swedish ethical-vetting authority, Dnr 2012/627 and 2017/813.

## Supplementary Material

dyac211_Supplementary_Data

## Data Availability

The data underlying this article cannot be shared publicly due to privacy concerns restricting availability of register data for research. Aggregated data can be made available by the authors, conditional on ethical vetting. The authors accessed the individual-level data through online access systems administered and hosted by each country’s central statistical agency.
