# Supplementary material for: Maternal age and the risk of low birthweight and pre-term delivery: a pan-Nordic comparison
Source: Int J Epidemiol. 2022 Nov 9;52(1):156–64. doi: 10.1093/ije/dyac211 (PMC9908063; doi:10.1093/ije/dyac211)
Supplement: dyac211_Supplementary_Data [file dyac211_supplementary_data.zip › dyac211_Supplementary_Data/ije-2021-10-1559-File005.docx]

**Figure S1. Mean age at maternal childbearing from Human Fertility Database for Denmark, Finland, Norway, and Sweden, 1980-2020.**


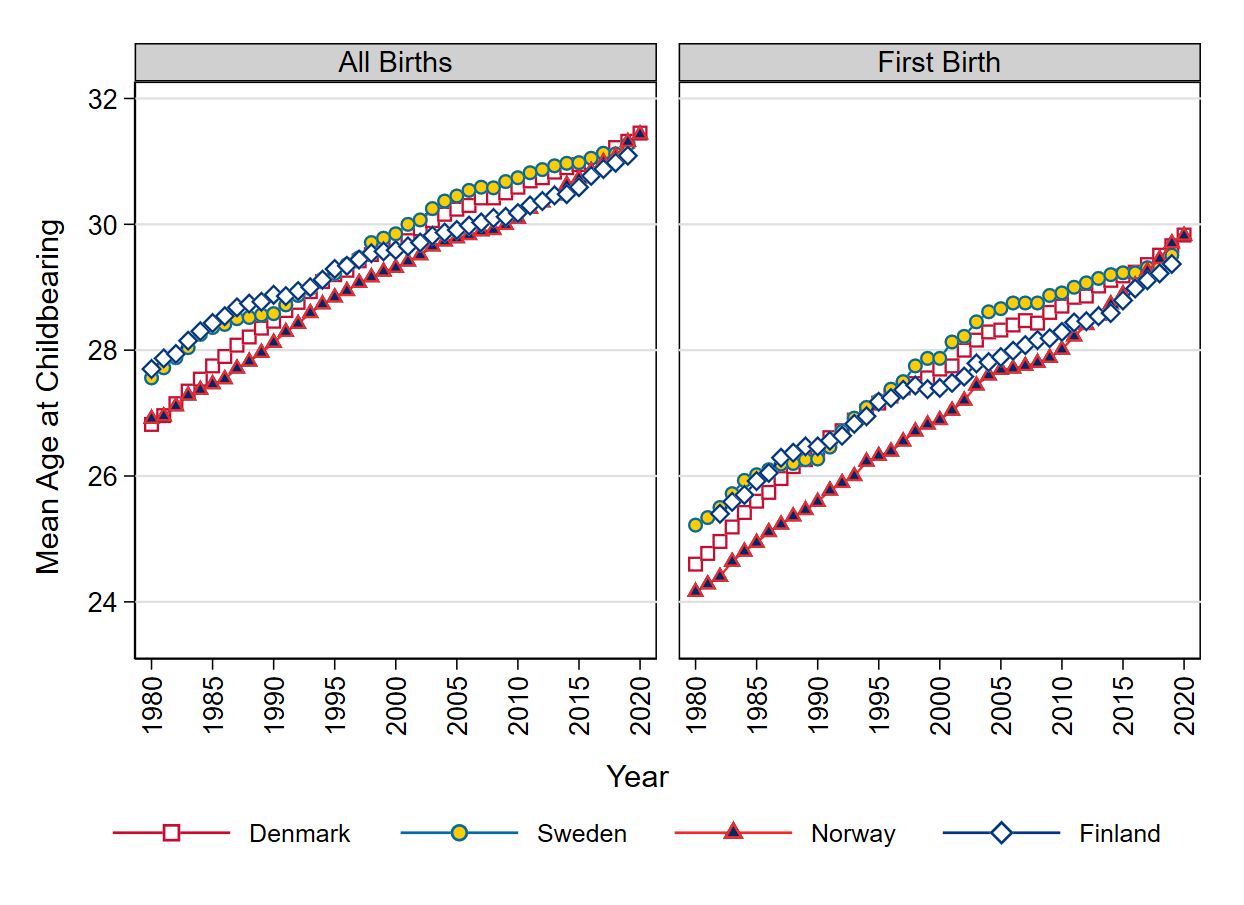


Source: Human Fertility Database (https://www.humanfertility.org).

Note: Period mean age at birth based on unconditional age-specific fertility rates for all birth orders combined. Detailed information on how this is calculated can be found on pages 41-42 of the Human Fertility Database methods document: <https://www.humanfertility.org/Docs/methods.pdf>

**Figure S2. Total fertility rates from Human Fertility Database for Denmark, Finland, Norway, and Sweden, 1980-2020.**

**
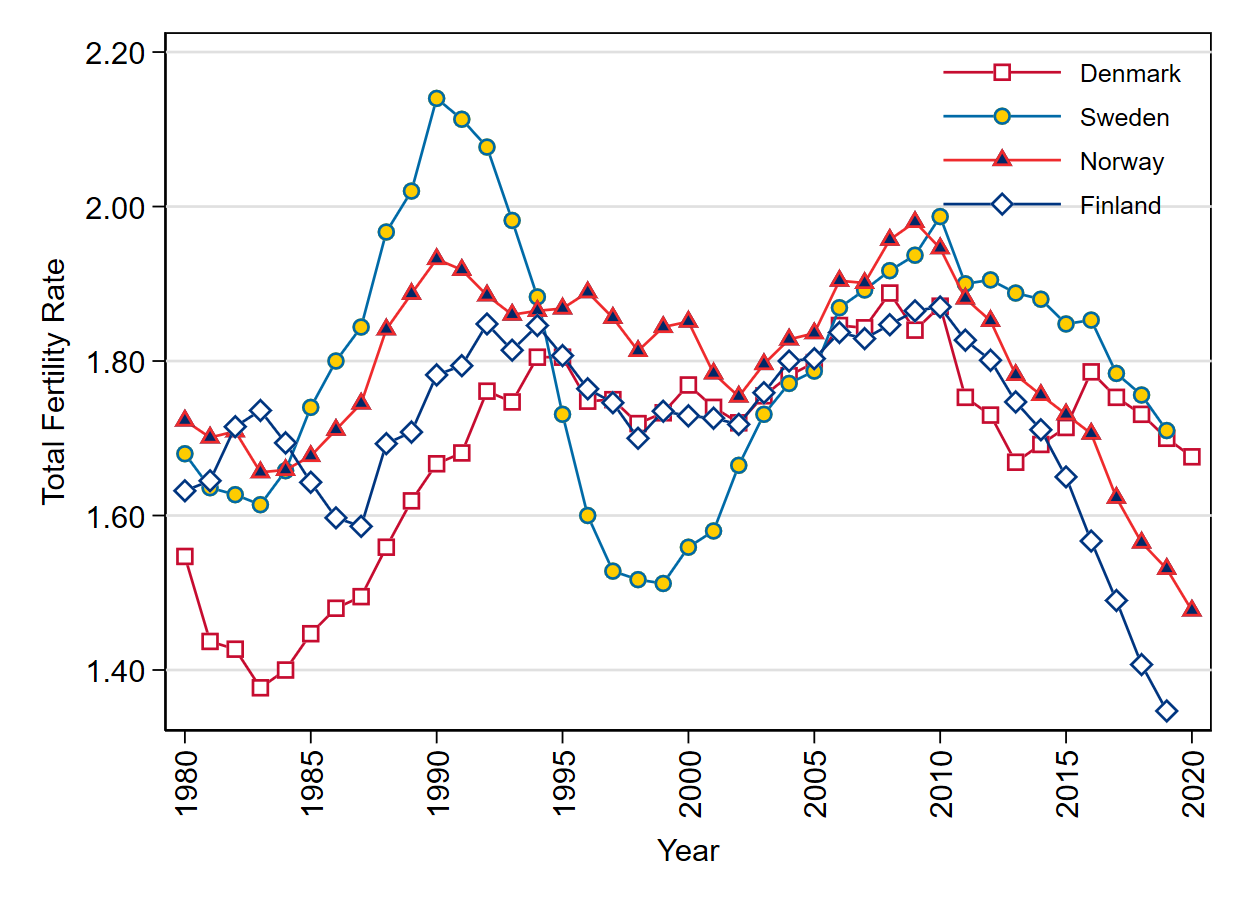
**

Source: Human Fertility Database (https://www.humanfertility.org).

Note: The Total Fertility Rate (TFR) is a period measure of fertility, in this case period total fertility rate based on unconditional age-specific fertility rates for all birth orders combined. Detailed information on how this is calculated can be found on pages 39-40 of the Human Fertility Database methods document: https://www.humanfertility.org/Docs/methods.pdf

**Figure S3. Completed cohort fertility from Human Fertility Database for Denmark, Finland, Norway, and Sweden, cohorts born 1920-1970.**

**
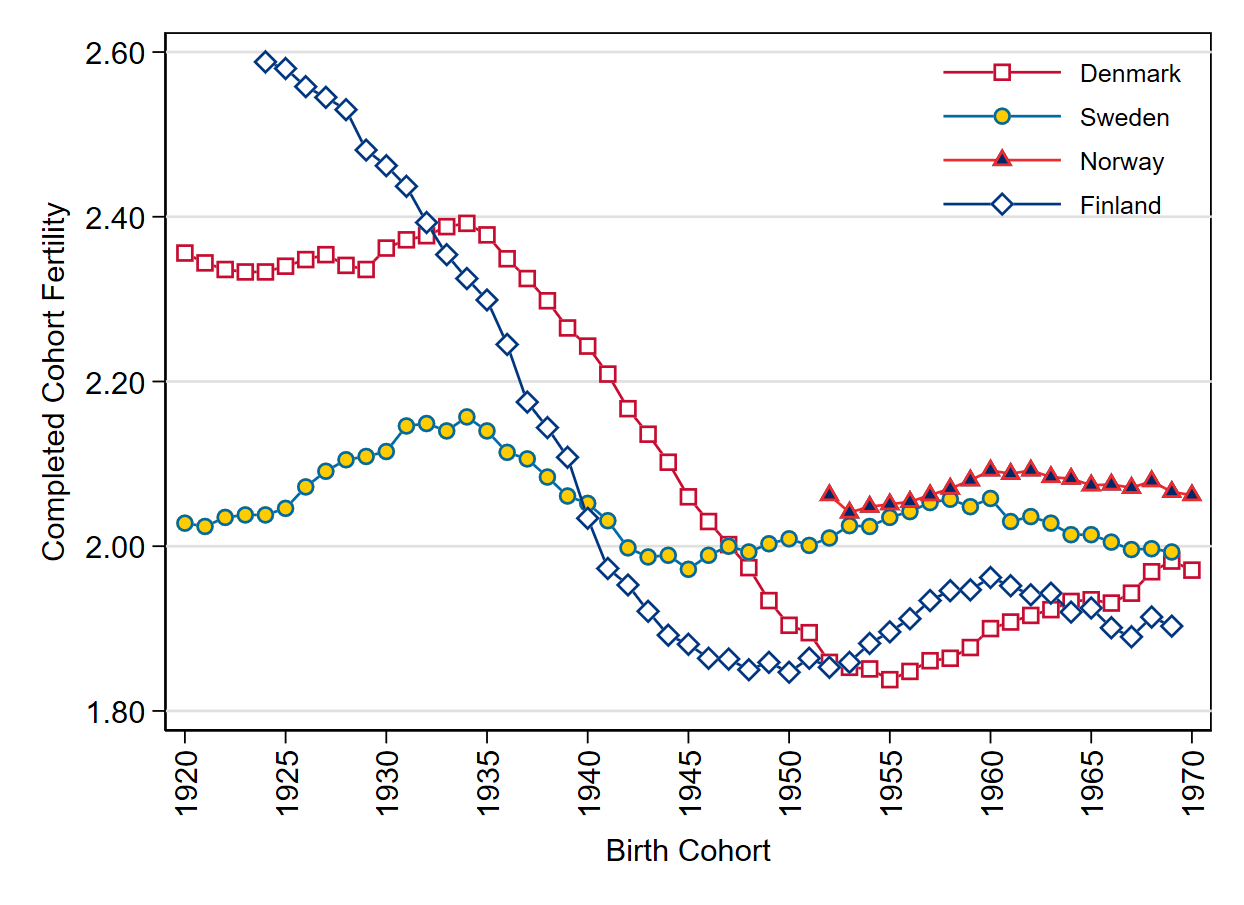
**

Source: Human Fertility Database (https://www.humanfertility.org).

Note: The Completed Cohort Fertility Rate (CCFR) is a cohort measure of fertility that reflects the actual number of children born to women over their life course, rather than a period-based approximation of fertility rates. Detailed information on how this is calculated can be found on pages 39-40 of the Human Fertility Database methods document: <https://www.humanfertility.org/Docs/methods.pdf>

**Figure S4: Prevalence of LBW and Preterm Birth in Sweden, Denmark and Norway during the period 1999-2012 and for Finland during the period 2001-2014**


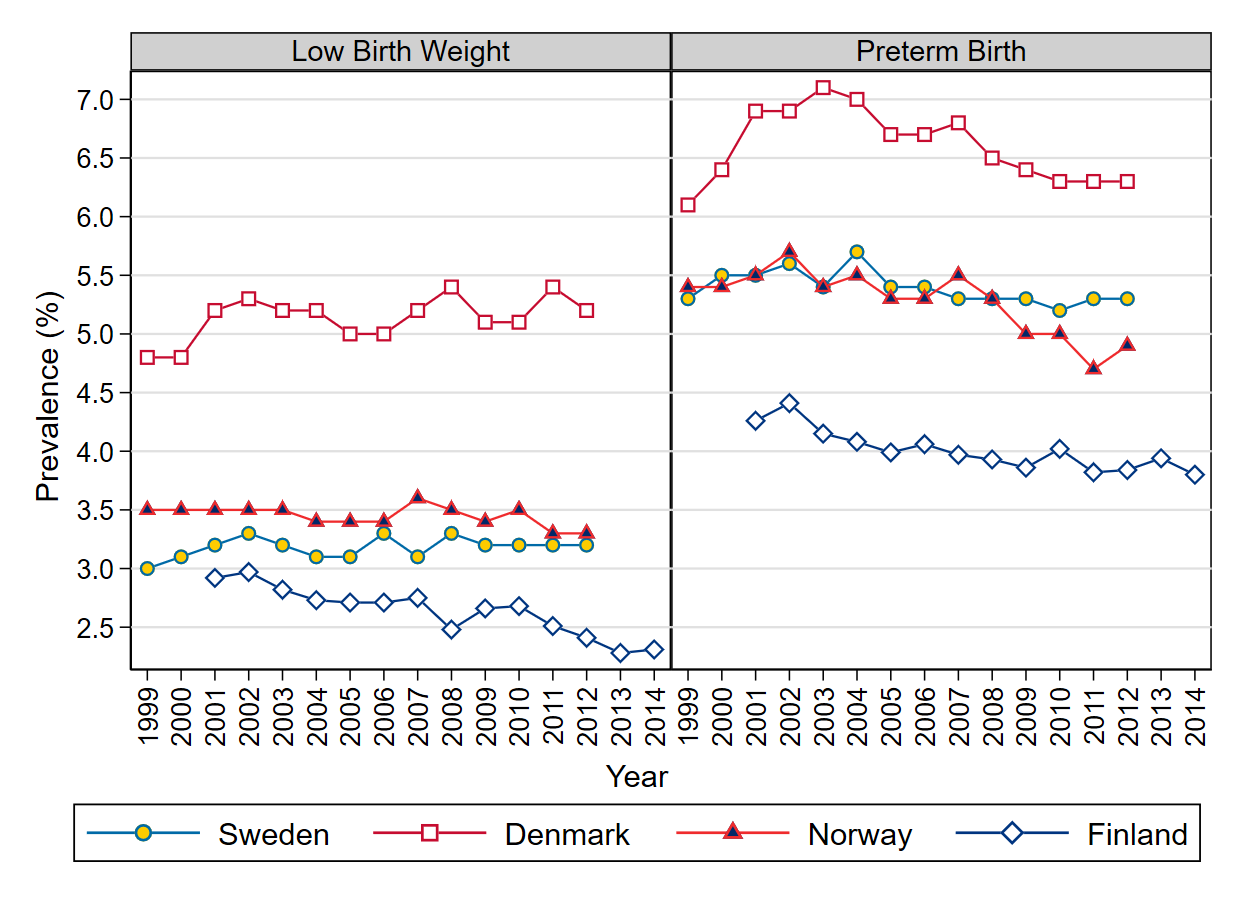


Source: Population register data, based on authors’ own calculations.

**Figure S5: The effect of maternal age on the risk of LBW from cousin comparison and sibling comparison approaches in Norway, children born 1999-2012.**


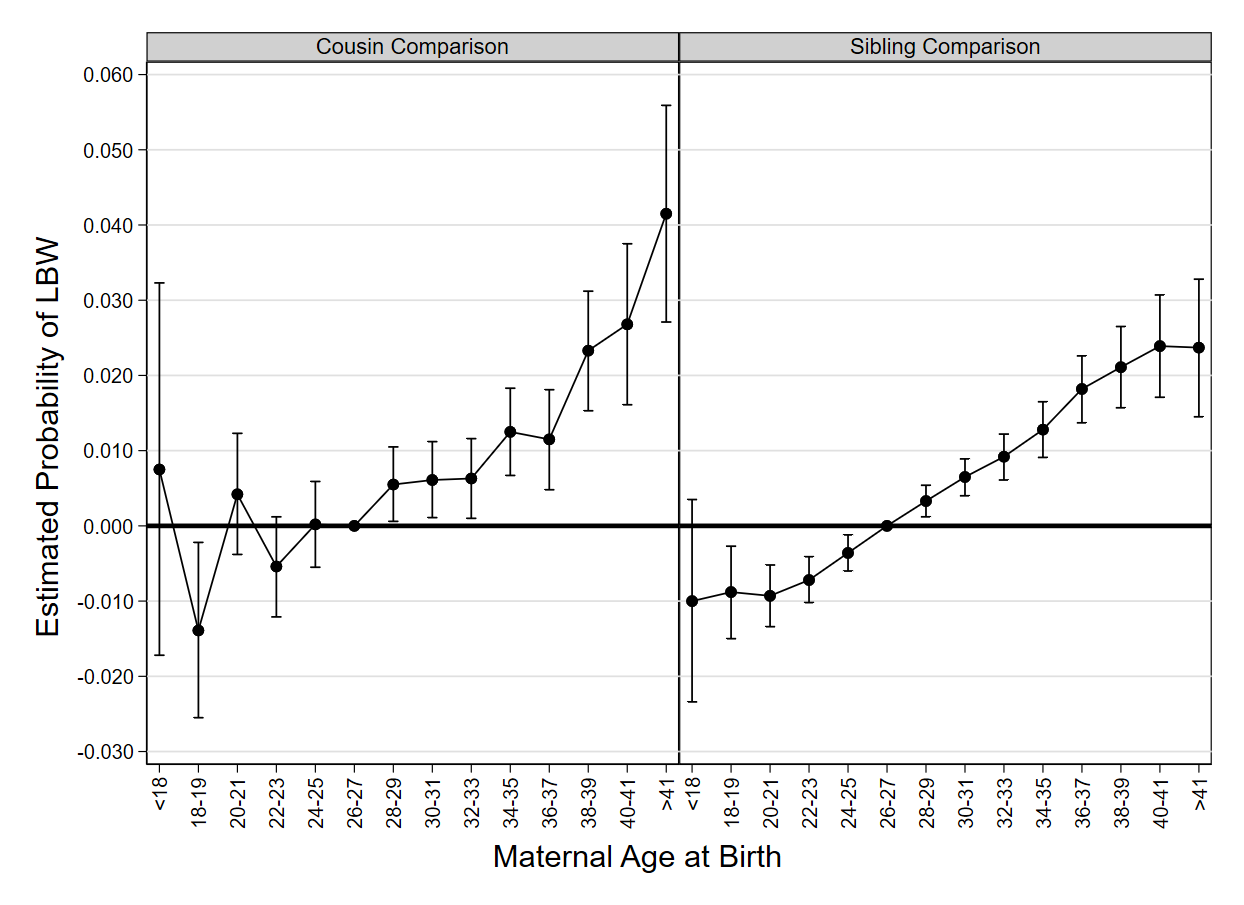


Source: Authors own calculations using Norwegian population register data.

Note: In this analysis the shared cousin group is defined by a shared maternal grandmother. In the cousin fixed effects analysis we focus on the earliest-born sibling in each sibling group born within the birth cohort range that we study, nested within the broader cousin group.
