# Supplementary material for: Maternal age and the risk of low birthweight and pre-term delivery: a pan-Nordic comparison
Source: Int J Epidemiol. 2022 Nov 9;52(1):156–64. doi: 10.1093/ije/dyac211 (PMC9908063; doi:10.1093/ije/dyac211)
Supplement: dyac211_Supplementary_Data [file dyac211_supplementary_data.zip › dyac211_Supplementary_Data/ije-2021-10-1559-File006.docx]

**Table S1. Model estimates from sibling fixed effects linear probability models for the association between maternal age and low birth weight and preterm delivery by country (estimates plotted in Figures 1 and 2).**

**Note: Model 0: No adjustment; Model 1: Adjustments for parity, and sex; Model 2: Model 1 + birth year.**

**Denmark**

| Low Birth Weight | | | | | | |
| --- | --- | --- | --- | --- | --- | --- |
|  | Model 0 |  | Model 1 |  | Model 2 |  |
| Age groups | Prob. diff. | 95% CI | Prob. diff. | 95% CI | Prob. diff. | 95% CI |
| <18 | 0.024 | 0.013, 0.034 | 0.018 | 0.003, 0.034 | 0.002 | -0.009, 0.012 |
| 18-19 | 0.015 | 0.010, 0.021 | 0.001 | -0.010, 0.011 | -0.012 | -0.018, -0.007 |
| 20-21 | 0.010 | 0.007, 0.014 | -0.001 | -0.009, 0.006 | -0.011 | -0.015, -0.007 |
| 22-23 | 0.005 | 0.002, 0.008 | -0.003 | -0.008, 0.003 | -0.009 | -0.012, -0.006 |
| 24-25 | 0.004 | 0.002, 0.006 | -0.001 | -0.004, 0.002 | -0.004 | -0.006, -0.002 |
| 26-27 | Ref. |  | Ref. |  | Ref. |  |
| 28-29 | -0.006 | -0.008, -0.004 | -0.001 | -0.004, 0.002 | 0.002 | 0.000, 0.004 |
| 30-31 | -0.010 | -0.012, -0.008 | -0.000 | -0.005, 0.005 | 0.006 | 0.004, 0.008 |
| 32-33 | -0.014 | -0.016, -0.012 | -0.001 | -0.008, 0.007 | 0.009 | 0.006, 0.012 |
| 34-35 | -0.014 | -0.016, -0.011 | 0.002 | -0.008, 0.011 | 0.014 | 0.011, 0.018 |
| 36-37 | -0.016 | -0.019, -0.013 | 0.002 | -0.010, 0.013 | 0.017 | 0.013, 0.022 |
| 38-39 | -0.016 | -0.020, -0.012 | 0.003 | -0.012, 0.017 | 0.022 | 0.016, 0.027 |
| 40-41 | -0.016 | -0.022, -0.010 | 0.004 | -0.013, 0.021 | 0.026 | 0.018, 0.033 |
| 42+ | -0.012 | -0.022, -0.001 | 0.008 | -0.013, 0.029 | 0.034 | 0.022, 0.045 |
| Preterm Delivery | | | | | | |
|  | Model 0 |  | Model 1 |  | Model 2 |  |
| Age groups | Prob. diff. | 95% CI | Prob. diff. | 95% CI | Prob. diff. | 95% CI |
| <18 | 0.028 | 0.016, 0.040 | 0.015 | -0.004, 0.033 | 0.002 | -0.011, 0.014 |
| 18-19 | 0.019 | 0.012, 0.025 | -0.002 | -0.014,-0.010 | -0.012 | -0.019,-0.006 |
| 20-21 | 0.012 | 0.007, 0.016 | -0.004 | -0.013, 0.005 | -0.012 | -0.016,-0.007 |
| 22-23 | 0.009 | 0.006, 0.013 | -0.001 | -0.007, 0.005 | -0.006 | -0.010,-0.003 |
| 24-25 | 0.008 | 0.005, 0.011 | 0.002 | -0.002, 0.006 | 0.000 | -0.003, 0.002 |
| 26-27 | Ref. |  | Ref. |  | Ref. |  |
| 28-29 | -0.009 | -0.011, -0.006 | -0.002 | -0.005, 0.002 | 0.001 | -0.002, 0.003 |
| 30-31 | -0.016 | -0.018, -0.013 | -0.003 | -0.009, 0.003 | 0.002 | -0.001, 0.005 |
| 32-33 | -0.020 | -0.024, -0.017 | -0.002 | -0.011, 0.006 | 0.005 | 0.001, 0.008 |
| 34-35 | -0.023 | -0.026, -0.020 | -0.001 | -0.013, 0.010 | 0.008 | 0.004, 0.012 |
| 36-37 | -0.020 | -0.024, -0.017 | 0.004 | -0.010, 0.018 | 0.016 | 0.011, 0.021 |
| 38-39 | -0.021 | -0.026, -0.016 | 0.006 | -0.011, 0.022 | 0.020 | 0.013, 0.026 |
| 40-41 | -0.020 | -0.027, -0.012 | 0.009 | -0.011, 0.029 | 0.025 | 0.017, 0.034 |
| 42+ | -0.015 | -0.027, -0.002 | 0.015 | -0.010, 0.040 | 0.034 | 0.021, 0.048 |

**Finland**

| Low Birth Weight | | | | | | |
| --- | --- | --- | --- | --- | --- | --- |
|  | Model 0 |  | Model 1 |  | Model 2 |  |
| Age groups | Prob. diff. | 95% CI | Prob. diff. | 95% CI | Prob. diff. | 95% CI |
| <18 | 0.026 | 0.018, 0.035 | -0.005 | -0.014, 0.004 | 0.013 | 0.002, 0.024 |
| 18-19 | 0.013 | 0.009, 0.017 | -0.012 | -0.017, -0.007 | 0.002 | -0.005, 0.009 |
| 20-21 | 0.010 | 0.007, 0.012 | -0.007 | -0.011, -0.004 | 0.004 | -0.002, 0.009 |
| 22-23 | 0.003 | 0.001, 0.006 | -0.006 | -0.009, -0.004 | 0.001 | -0.003, 0.005 |
| 24-25 | 0.003 | 0.001, 0.005 | -0.002 | -0.004, 0.000 | 0.002 | -0.001, 0.004 |
| 26-27 | Ref. |  | Ref. |  | Ref. |  |
| 28-29 | -0.003 | -0.005, -0.001 | 0.002 | 0.000, 0.004 | -0.001 | -0.004, 0.001 |
| 30-31 | -0.006 | -0.007, -0.004 | 0.004 | 0.002, 0.006 | -0.003 | -0.007, 0.000 |
| 32-33 | -0.009 | -0.011, -0.007 | 0.005 | 0.002, 0.008 | -0.006 | -0.011, -0.001 |
| 34-35 | -0.011 | -0.013, -0.008 | 0.007 | 0.003, 0.010 | -0.008 | -0.015, -0.002 |
| 36-37 | -0.011 | -0.013, -0.008 | 0.010 | 0.006, 0.013 | -0.009 | -0.017, -0.001 |
| 38-39 | -0.014 | -0.017, -0.010 | 0.009 | 0.005, 0.014 | -0.013 | -0.022, -0.003 |
| 40-41 | -0.012 | -0.016, -0.007 | 0.013 | 0.008, 0.019 | -0.012 | -0.024, -0.001 |
| 42+ | -0.006 | -0.013, 0.000 | 0.020 | 0.013, 0.028 | -0.010 | -0.024, 0.004 |
| Preterm Delivery | | | | | | |
|  | Model 0 |  | Model 1 |  | Model 2 |  |
| Age groups | Prob. diff. | 95% CI | Prob. diff. | 95% CI | Prob. diff. | 95% CI |
| <18 | 0.021 | 0.010, 0.031 | -0.010 | -0.021, 0.001 | 0.006 | -0.007, 0.020 |
| 18-19 | 0.006 | 0.001, 0.011 | -0.018 | -0.023, -0.012 | -0.005 | -0.014, 0.004 |
| 20-21 | 0.005 | 0.002, 0.008 | -0.011 | -0.015, -0.007 | -0.001 | -0.008, 0.005 |
| 22-23 | 0.001 | -0.001, 0.004 | -0.008 | -0.011, -0.004 | -0.001 | -0.006, 0.004 |
| 24-25 | 0.003 | 0.001, 0.005 | -0.001 | -0.004, 0.001 | 0.002 | -0.001, 0.005 |
| 26-27 | Ref. |  | Ref. |  | Ref. |  |
| 28-29 | -0.003 | -0.005, 0.000 | 0.002 | 0.000, 0.004 | -0.001 | -0.004, 0.002 |
| 30-31 | -0.007 | -0.010, -0.005 | 0.002 | -0.001, 0.005 | -0.005 | -0.009, -0.001 |
| 32-33 | -0.009 | -0.011, -0.006 | 0.004 | 0.001, 0.008 | -0.006 | -0.012, 0.000 |
| 34-35 | -0.008 | -0.011, -0.005 | 0.009 | 0.005, 0.013 | -0.005 | -0.013, 0.003 |
| 36-37 | -0.006 | -0.009, -0.003 | 0.013 | 0.009, 0.018 | -0.004 | -0.014, 0.006 |
| 38-39 | -0.007 | -0.011, -0.003 | 0.014 | 0.009, 0.020 | -0.007 | -0.019, 0.005 |
| 40-41 | -0.001 | -0.007, 0.005 | 0.022 | 0.015, 0.029 | -0.002 | -0.016, 0.012 |
| 42+ | 0.006 | -0.002, 0.014 | 0.030 | 0.021, 0.039 | 0.001 | -0.016, 0.018 |

**Norway**

| Low Birth Weight | | | | | | |
| --- | --- | --- | --- | --- | --- | --- |
|  | Model 0 |  | Model 1 | | Model 2 |  |
| Age groups | Prob. diff. | 95% CI | Prob. diff. | 95% CI | Prob. diff. | 95% CI |
| <18 | 0.032 | 0.019, 0.045 | -0.010 | -0.023, 0.003 | 0.001 | -0.016, 0.018 |
| 18-19 | 0.026 | 0.020, 0.031 | -0.009 | -0.015, -0.003 | 0.000 | -0.010, 0.011 |
| 20-21 | 0.016 | 0.013, 0.020 | -0.009 | -0.013, -0.005 | -0.003 | -0.010, 0.005 |
| 22-23 | 0.009 | 0.006, 0.012 | -0.007 | -0.010, -0.004 | -0.003 | -0.008, 0.003 |
| 24-25 | 0.004 | 0.002, 0.006 | -0.004 | -0.006, -0.001 | -0.001 | -0.004, 0.002 |
| 26-27 | Ref. |  | Ref. |  | Ref. |  |
| 28-29 | -0.004 | -0.006, -0.003 | 0.003 | 0.001, 0.005 | 0.001 | -0.002, 0.004 |
| 30-31 | -0.009 | -0.011, -0.007 | 0.006 | 0.004, 0.009 | 0.002 | -0.003, 0.007 |
| 32-33 | -0.014 | -0.016, -0.011 | 0.009 | 0.006, 0.012 | 0.002 | -0.005, 0.009 |
| 34-35 | -0.016 | -0.019, -0.014 | 0.013 | 0.009, 0.017 | 0.004 | -0.005, 0.013 |
| 36-37 | -0.016 | -0.019, -0.013 | 0.018 | 0.014, 0.023 | 0.007 | -0.005, 0.018 |
| 38-39 | -0.019 | -0.022, -0.015 | 0.021 | 0.016, 0.026 | 0.007 | -0.006, 0.021 |
| 40-41 | -0.021 | -0.026, -0.015 | 0.024 | 0.017, 0.031 | 0.008 | -0.008, 0.024 |
| 42+ | -0.025 | -0.033, -0.017 | 0.024 | 0.015, 0.033 | 0.005 | -0.015, 0.025 |
| Preterm Delivery | | | | | | |
|  | Model 0 |  | Model 1 | | Model 2 |  |
| Age groups | Prob. diff. | 95% CI | Prob. diff. | 95% CI | Prob. diff. | 95% CI |
| <18 | 0.064 | 0.048, 0.081 | 0.024 | 0.007, 0.041 | 0.001 | -0.016, 0.018 |
| 18-19 | 0.024 | 0.017, 0.031 | -0.009 | -0.017, -0.002 | 0.000 | -0.010, 0.011 |
| 20-21 | 0.019 | 0.014, 0.023 | -0.005 | -0.011, 0.000 | -0.003 | -0.010, 0.005 |
| 22-23 | 0.008 | 0.004, 0.011 | -0.007 | -0.011, -0.003 | -0.003 | -0.008, 0.003 |
| 24-25 | 0.005 | 0.003, 0.008 | -0.002 | -0.005, -0.001 | -0.001 | -0.005, 0.002 |
| 26-27 | Ref. |  | Ref. |  | Ref. |  |
| 28-29 | -0.004 | -0.007, -0.002 | 0.003 | 0.000, 0.005 | -0.001 | -0.002, 0.004 |
| 30-31 | -0.010 | -0.013, -0.008 | 0.004 | 0.001, 0.007 | -0.002 | -0.003, 0.007 |
| 32-33 | -0.014 | -0.016, -0.011 | 0.007 | 0.003, 0.011 | -0.003 | -0.005, 0.009 |
| 34-35 | -0.016 | -0.020, -0.013 | 0.009 | 0.005, 0.014 | 0.004 | -0.005, 0.013 |
| 36-37 | -0.015 | -0.019, -0.012 | 0.015 | 0.010, 0.021 | 0.007 | -0.005, 0.018 |
| 38-39 | -0.019 | -0.023, -0.014 | 0.016 | 0.009, 0.023 | 0.008 | -0.006, 0.021 |
| 40-41 | -0.015 | -0.021, -0.008 | 0.024 | 0.015, 0.032 | 0.008 | -0.008, 0.024 |
| 42+ | -0.015 | -0.025, -0.006 | 0.026 | 0.015, 0.038 | 0.005 | -0.015, 0.025 |

**Sweden**

| Low Birth Weight | | | | | | |
| --- | --- | --- | --- | --- | --- | --- |
|  | Model 0 |  | Model 1 |  | Model 2 |  |
| Age groups | Prob. diff. | 95% CI | Prob. diff. | 95% CI | Prob. diff. | 95% CI |
| <18 | 0.035 | 0.024, 0.045 | 0.001 | -0.013, 0.015 | -0.016 | -0.027, -0.006 |
| 18-19 | 0.019 | 0.014, 0.024 | -0.009 | -0.018, 0.000 | -0.022 | -0.028, -0.017 |
| 20-21 | 0.016 | 0.013, 0.019 | -0.005 | -0.012, 0.001 | -0.016 | -0.019, -0.012 |
| 22-23 | 0.008 | 0.006, 0.010 | -0.005 | -0.009, -0.001 | -0.012 | -0.015, -0.010 |
| 24-25 | 0.004 | 0.002, 0.006 | -0.002 | -0.004, 0.001 | -0.005 | -0.007, -0.003 |
| 26-27 | Ref. |  | Ref. |  |  |  |
| 28-29 | -0.006 | -0.007, -0.004 | 0.000 | -0.002, 0.003 | 0.004 | 0.002, 0.005 |
| 30-31 | -0.009 | -0.011, -0.008 | 0.002 | -0.002, 0.006 | 0.009 | 0.007, 0.011 |
| 32-33 | -0.014 | -0.016, -0.013 | 0.003 | -0.002, 0.009 | 0.014 | 0.011, 0.016 |
| 34-35 | -0.018 | -0.020, -0.016 | 0.005 | -0.002, 0.013 | 0.019 | 0.016, 0.022 |
| 36-37 | -0.020 | -0.022, -0.017 | 0.006 | -0.003, 0.016 | 0.024 | 0.020, 0.027 |
| 38-39 | -0.025 | -0.028, -0.022 | 0.003 | -0.009, 0.014 | 0.024 | 0.019, 0.028 |
| 40-41 | -0.021 | -0.026, -0.016 | 0.006 | -0.007, 0.020 | 0.031 | 0.025, 0.037 |
| 42+ | -0.016 | -0.023, -0.009 | 0.010 | -0.007, 0.026 | 0.038 | 0.030, 0.046 |
| Preterm Delivery | | | | | | |
|  | Model 0 |  | Model 1 |  | Model 2 |  |
| Age groups | Prob. diff. | 95% CI | Prob. diff. | 95% CI | Prob. diff. | 95% CI |
| <18 | 0.026 | 0.013, 0.039 | -0.015 | -0.033, 0.003 | -0.030 | -0.044, -0.017 |
| 18-19 | 0.019 | 0.012, 0.025 | -0.015 | -0.026, -0.004 | -0.027 | -0.034, -0.020 |
| 20-21 | 0.013 | 0.009, 0.017 | -0.012 | -0.020, -0.004 | 0.021 | -0.025, -0.016 |
| 22-23 | 0.010 | 0.008, 0.013 | -0.005 | -0.010, 0.001 | -0.011 | -0.014, -0.008 |
| 24-25 | 0.006 | 0.003, 0.008 | -0.001 | -0.005, 0.002 | -0.004 | -0.007, -0.002 |
| 26-27 | Ref. |  | Ref. |  | Ref. |  |
| 28-29 | -0.008 | -0.010, -0.006 | -0.001 | -0.004, 0.002 | 0.002 | 0.000, 0.004 |
| 30-31 | -0.015 | -0.017, -0.013 | -0.001 | -0.006, 0.004 | 0.005 | 0.002, 0.007 |
| 32-33 | -0.022 | -0.024, -0.020 | -0.001 | -0.008, 0.007 | 0.009 | 0.005, 0.012 |
| 34-35 | -0.023 | -0.026, -0.021 | 0.003 | -0.007, 0.013 | 0.016 | 0.011, 0.020 |
| 36-37 | -0.026 | -0.029, -0.022 | 0.004 | -0.008, 0.017 | 0.020 | 0.015, 0.025 |
| 38-39 | -0.027 | -0.031, -0.022 | 0.005 | -0.010, 0.019 | 0.024 | 0.018, 0.095 |
| 40-41 | -0.023 | -0.029, -0.017 | 0.007 | -0.010, 0.025 | 0.030 | 0.022, 0.037 |
| 42+ | -0.014 | -0.023, -0.004 | 0.014 | -0.007, 0.035 | 0.040 | 0.030, 0.051 |

**Table S2. Model estimates from sensitivity analyses on the association between maternal age and low birth weight and preterm delivery by country. Model 4 (inter-pregnancy interval analysis) only available for Norway and Finland.**

**Note: Model 3: Sibling FE models only adjusting for sex; Model 4: Sibling FE model adjusting for sex, birth order, and inter-pregnancy interval.**

**Denmark**

| Low Birth Weight | | |
| --- | --- | --- |
|  | Model 3 |  |
| Age groups | Prob. diff. | 95% CI |
| <18 | 0.024 | 0.013, 0.034 |
| 18-19 | 0.015 | 0.010, 0.021 |
| 20-21 | 0.010 | 0.007, 0.014 |
| 22-23 | 0.005 | 0.002, 0.008 |
| 24-25 | 0.004 | 0.002, 0.006 |
| 26-27 | Ref. |  |
| 28-29 | -0.006 | -0.008, -0.004 |
| 30-31 | -0.010 | -0.012, -0.008 |
| 32-33 | -0.014 | -0.016, -0.012 |
| 34-35 | -0.014 | -0.016, -0.011 |
| 36-37 | -0.016 | -0.019, -0.013 |
| 38-39 | -0.016 | -0.021, -0.012 |
| 40-41 | -0.016 | -0.023, -0.010 |
| 42+ | -0.012 | -0.022, -0.001 |
| Preterm Delivery | | |
|  | Model 3 |  |
| Age groups | Prob. diff. | 95% CI |
| <18 | 0.028 | 0.016, 0.040 |
| 18-19 | 0.019 | 0.012, 0.025 |
| 20-21 | 0.012 | 0.007, 0.016 |
| 22-23 | 0.009 | 0.006, 0.013 |
| 24-25 | 0.008 | 0.005, 0.011 |
| 26-27 | Ref. |  |
| 28-29 | -0.008 | -0.011, -0.006 |
| 30-31 | -0.016 | -0.018, -0.013 |
| 32-33 | -0.020 | -0.023, -0.018 |
| 34-35 | -0.023 | -0.026, -0.020 |
| 36-37 | -0.020 | -0.024, -0.017 |
| 38-39 | -0.021 | -0.026, -0.016 |
| 40-41 | -0.020 | -0.027, -0.012 |
| 42+ | -0.014 | -0.027, -0.002 |

**Finland**

| Low birthweight | | | | |
| --- | --- | --- | --- | --- |
|  | Model 3 |  | Model 4 | |
| Age groups | Prob. diff. | 95% CI | Prob. diff. | 95% CI |
| <18 | 0.026 | 0.018, 0.035 | -0.000 | -0.010, 0.009 |
| 18-19 | 0.013 | 0.009, 0.017 | -0.009 | -0.013, -0.004 |
| 20-21 | 0.010 | 0.007, 0.012 | -0.005 | -0.008, -0.001 |
| 22-23 | 0.003 | 0.001, 0.006 | -0.004 | -0.007, -0.002 |
| 24-25 | 0.003 | 0.001, 0.005 | -0.001 | -0.003, 0.001 |
| 26-27 | Ref. |  | Ref. |  |
| 28-29 | -0.003 | -0.005, -0.001 | 0.001 | -0.001, 0.003 |
| 30-31 | -0.006 | -0.007, -0.004 | 0.002 | 0.000, 0.005 |
| 32-33 | -0.009 | -0.011, -0.007 | 0.002 | -0.001, 0.005 |
| 34-35 | -0.011 | -0.013, -0.008 | 0.003 | -0.000, 0.007 |
| 36-37 | -0.011 | -0.014, -0.008 | 0.005 | 0.001, 0.010 |
| 38-39 | -0.014 | -0.017, -0.010 | 0.004 | -0.001, 0.009 |
| 40-41 | -0.012 | -0.016, -0.007 | 0.008 | 0.002, 0.014 |
| 42+ | -0.006 | -0.013, 0.000 | 0.014 | 0.006, 0.022 |
| Preterm Delivery | | | | |
|  | Model 3 |  | Model 4 | |
| Age groups | Prob. diff. | 95% CI | Prob. diff. | 95% CI |
| <18 | 0.020 | 0.010, 0.031 | -0.010 | -0.021, 0.001 |
| 18-19 | 0.006 | 0.001, 0.011 | -0.018 | -0.024, -0.012 |
| 20-21 | 0.005 | 0.002, 0.008 | -0.012 | -0.016, -0.008 |
| 22-23 | 0.001 | -0.001, 0.004 | -0.008 | -0.011, -0.005 |
| 24-25 | 0.003 | 0.001, 0.005 | -0.002 | -0.004, 0.001 |
| 26-27 | Ref. |  | Ref. |  |
| 28-29 | -0.003 | -0.005, -0.000 | 0.002 | 0.000, 0.005 |
| 30-31 | -0.007 | -0.010, -0.005 | 0.002 | -0.001, 0.005 |
| 32-33 | -0.009 | -0.011, -0.006 | 0.005 | 0.001, 0.008 |
| 34-35 | -0.008 | -0.011, -0.005 | 0.009 | 0.005, 0.013 |
| 36-37 | -0.006 | -0.009, -0.002 | 0.014 | 0.009, 0.019 |
| 38-39 | -0.007 | -0.011, -0.003 | 0.015 | 0.009, 0.021 |
| 40-41 | -0.001 | -0.007, 0.005 | 0.023 | 0.015, 0.030 |
| 42+ | 0.006 | -0.002, 0.014 | 0.031 | 0.021, 0.040 |

**Norway**

| Low birthweight | | | | |
| --- | --- | --- | --- | --- |
|  | Model 3 |  | Model 4 | |
| Age groups | Prob. diff. | 95% CI | Prob. diff. | 95% CI |
| <18 | 0.032 | 0.019, 0.045 | -0.005 | -0.019, 0.009 |
| 18-19 | 0.026 | 0.020, 0.031 | -0.005 | -0.012, 0.002 |
| 20-21 | 0.016 | 0.013, 0.019 | -0.006 | -0.011, -0.002 |
| 22-23 | 0.009 | 0.006, 0.012 | -0.005 | -0.008, -0.002 |
| 24-25 | 0.004 | 0.002, 0.006 | -0.002 | -0.005, 0.000 |
| 26-27 | Ref |  | Ref |  |
| 28-29 | -0.004 | -0.006, -0.003 | 0.002 | 0.000, 0.004 |
| 30-31 | -0.009 | -0.011, -0.007 | 0.004 | 0.001, 0.007 |
| 32-33 | -0.014 | -0.016, -0.011 | 0.006 | 0.002, 0.010 |
| 34-35 | -0.016 | -0.019, -0.014 | 0.009 | 0.004, 0.013 |
| 36-37 | -0.016 | -0.019, -0.014 | 0.013 | 0.007, 0.018 |
| 38-39 | -0.019 | -0.023, -0.015 | 0.015 | 0.008, 0.022 |
| 40-41 | -0.021 | -0.026, -0.016 | 0.017 | 0.009, 0.025 |
| 42+ | -0.025 | -0.033, -0.017 | 0.016 | 0.006, 0.026 |
| Preterm Delivery | | | | |
|  | Model 3 |  | Model 4 | |
| Age groups | Prob. diff. | 95% CI | Prob. diff. | 95% CI |
| <18 | 0.064 | 0.048, 0.080 | 0.023 | 0.006, 0.040 |
| 18-19 | 0.024 | 0.017, 0.031 | -0.010 | -0.019, -0.002 |
| 20-21 | 0.019 | 0.014, 0.023 | -0.006 | -0.012, -0.001 |
| 22-23 | 0.008 | 0.004, 0.011 | -0.008 | -0.012, -0.003 |
| 24-25 | 0.005 | 0.002, 0.008 | -0.002 | -0.005, 0.001 |
| 26-27 | Ref |  | Ref |  |
| 28-29 | -0.004 | -0.007, -0.002 | 0.003 | 0.000, 0.006 |
| 30-31 | -0.010 | -0.013, -0.008 | 0.004 | 0.001, 0.008 |
| 32-33 | -0.013 | -0.016, -0.011 | 0.008 | 0.003, 0.013 |
| 34-35 | -0.016 | -0.020, -0.013 | 0.011 | 0.005, 0.016 |
| 36-37 | -0.015 | -0.019, -0.012 | 0.017 | 0.010, 0.024 |
| 38-39 | -0.018 | -0.023, -0.014 | 0.018 | 0.009, 0.026 |
| 40-41 | -0.014 | -0.021, -0.008 | 0.026 | 0.016, 0.036 |
| 42+ | -0.015 | -0.025, -0.006 | 0.029 | 0.016, 0.042 |

**Sweden**

| Low Birth Weight | | |
| --- | --- | --- |
|  | Model 3 |  |
| Age groups | Prob. diff. | 95% CI |
| <18 | 0.035 | 0.024, 0.045 |
| 18-19 | 0.019 | 0.014, 0.024 |
| 20-21 | 0.016 | 0.013, 0.018 |
| 22-23 | 0.008 | 0.006, 0.0099 |
| 24-25 | 0.004 | 0.002, 0.006 |
| 26-27 | Ref. |  |
| 28-29 | -0.006 | -0.007, -0.004 |
| 30-31 | -0.009 | -0.011, - 0.008 |
| 32-33 | -0.014 | -0.016, -0.013 |
| 34-35 | -0.018 | -0.0198, -0.016 |
| 36-37 | -0.0198 | -0.022, -0.017 |
| 38-39 | -0.025 | -0.028, -0.022 |
| 40-41 | -0.021 | -0.026, -0.016 |
| 42+ | -0.016 | -0.023, -0.009 |
| Preterm Delivery | | |
|  | Model 3 |  |
| Age groups | Prob. diff. | 95% CI |
| <18 | 0.026 | 0.013, 0.039 |
| 18-19 | 0.019 | 0.012, 0.025 |
| 20-21 | 0.013 | 0.0095, 0.017 |
| 22-23 | 0.010 | 0.007, 0.013 |
| 24-25 | 0.005 | 0.003, 0.008 |
| 26-27 | Ref. |  |
| 28-29 | -0.008 | -0.010, -0.006 |
| 30-31 | -0.015 | -0.017, -0.013 |
| 32-33 | -0.022 | -0.024, -0.019 |
| 34-35 | -0.023 | -0.026, -0.021 |
| 36-37 | -0.026 | -0.029, -0.022 |
| 38-39 | -0.026 | -0.031, -0.022 |
| 40-41 | -0.023 | -0.029, -0.017 |
| 42+ | -0.013 | -0.022, -0.004 |
